# Consumption-portfolio choice with subsistence consumption and risk aversion change at retirement

**DOI:** 10.1186/s13660-018-1756-1

**Published:** 2018-07-06

**Authors:** Ho-Seok Lee

**Affiliations:** Department of Mathematics, Kwangwoon University, Seoul, Republic of Korea

**Keywords:** Dynamic programming method, Free boundary value problem, Optimal stopping time, Risk aversion change, Subsistence consumption

## Abstract

This paper considers subsistence consumption of an economic agent both before and after retirement in analyzing the optimal consumption, portfolio, and retirement problem. We allow the relative risk aversion of the economic agent to make a one-off jump at retirement. With a Cobb–Douglas utility function, we obtain explicit expressions for the optimal policies. Numerical results show that, whereas post-retirement subsistence consumption tends to delay retirement, pre-retirement subsistence consumption and the magnitude of jump in relative risk aversion may stimulate early retirement. Also, the consumption drop at retirement deepens as post-retirement subsistence consumption increases, but it weakens as pre-retirement subsistence consumption increases.

## Introduction

In the present paper, we study lifetime consumption, portfolio, and retirement of an economic agent whose relative risk aversion changes at retirement, with the assumption that the agent faces both pre- and post-retirement subsistence consumption. Recently, Lim et al. [17] also considered both pre- and post-retirement subsistence consumption, but they did not consider risk aversion change at retirement. Also, our study and that of Lim et al. [17] are methodologically different: we use a dynamic programming method, whereas Lim et al. [17] relied on the martingale and duality approach. Risk aversion change at retirement has been well addressed in empirical studies (e.g., Yoo [23], Riley and Chow [20], and Halek and Eisenhauer [8]). According to the results of the above studies, risk aversion tends to increase substantially at retirement. Kwak et al. [13] considered risk aversion change at retirement, but did not impose any constraints (e.g., subsistence consumption constraint or borrowing constraint). Jang and Lee [10] investigated the combined effects of risk aversion change at retirement and borrowing constraints on the optimal consumption, portfolio, and retirement strategies. We allow the relative risk aversion of the economic agent to make a one-off jump at retirement as in Jang and Lee [10]. In addition, we assume a Cobb–Douglas utility function of consumption and leisure because this function is appropriate for capturing empirical observations that consumption drops significantly at retirement (for the *consumption retirement puzzle*: see Hurd and Rohwedder [9], Haider and Stephens [7], and Aguila et al. [1]).

This paper is an extension of consumption-portfolio choice literature that examines quantitatively the combined effects of subsistence consumption and risk aversion change at retirement. Merton’s [19] original problem on the optimal consumption and portfolio was generalized by Karatzas et al. [12], who transformed the relevant Bellman equation to a linear ordinary differential equation, which is more tractable than the original. Sethi et al. [21] extended Karatzas et al. [12] to the case with subsistence consumption, and exploited a different transformation to linearize the relevant Bellman equation. Shin et al. [22] used the Martingale and duality method to explore a similar problem. Lakner and Nygren [14] used Malliavin calculus to solve the portfolio optimization problem with subsistence consumption. Labor supply flexibility was firstly incorporated into lifetime consumption and portfolio selection problem by Bodie et al. [3]. Choi and Shim [4] and Farhi and Panageas [6] developed a more realistic model on labor supply flexibility through investigating the optimal retirement problem as an optimal stopping problem. Lim et al. [18] and Lee and Shin [15] considered the disutility from labor to extend these ideas that include subsistence consumption. Assuming a trade-off between income and leisure, Lee and Shin [16] studied the optimal consumption, portfolio, and retirement problem of an economic agent with a subsistence consumption. Most similar work to ours is by Lim et al. [17] and Lee and Shin [16], but they did not consider risk aversion change nor investigate the consumption drop at retirement. Restrictions on consumption level and limited access to credit market are realistic assumptions when we investigate lifetime optimal consumption and portfolio selection problem. Other than these constraints, individual’s uninsurable income risk or incomplete market are of crucial importance for studying lifetime optimal strategies. For example, Jang et al. [11] and Bensoussan et al. [2] developed optimal retirement rules of an individual with an exogenous unemployment risk.

Our numerical results show that pre-retirement subsistence consumption may urge early retirement, whereas post-retirement subsistence consumption may delay retirement. The wealth accumulation for retirement is likely to decrease with the magnitude of jump in relative risk aversion at retirement. We also investigate the effects of subsistence consumption on consumption drop at retirement if the relative risk aversion changes at retirement. Post-retirement subsistence consumption may intensify consumption drop at retirement, whereas pre-retirement subsistence consumption may weaken it. The remainder of this paper proceeds as follows. Section 2 introduces the financial market and constructs our model. Section 3 solves the economic agent’s optimization problem using dynamic programming method and obtains analytic expressions for the optimal policies. Some numerical illustrations and their implications are given in Sect. 4, and Sect. 5 summarizes the results.

## The model

In this paper, it is assumed that an infinitely-lived economic agent faces both pre- and post-retirement subsistence consumption. The economic agent has an option to retire (i.e., can choose retirement time) and retirement is irreversible. Let $R_{1}$ and $R_{2}$ be the pre-retirement subsistence consumption and post-retirement subsistence consumption, respectively. So, if we denote by $c_{t}$ the consumption rate process at time *t* and by *τ* the retirement time, the following inequality for the consumption rate must hold:

2.1 $$ c_{t} \geq R_{1}\quad \mbox{for all }0\leq t< \tau , c_{t}\geq R_{2} \mbox{ for all } t\geq \tau . $$

An economic agent derives utility from consumption and leisure. We use a Cobb–Douglas utility function of consumption and leisure with the form

2.2 $$ u(c_{t}, l_{t})\triangleq \frac{1}{\alpha } \cdot \frac{(c_{t}^{\alpha}l_{t}^{1-\alpha })^{1-\bar{\gamma }_{t}}}{1-\bar{\gamma }_{t}},\quad 0< \alpha < 1,\bar{\gamma }_{t}>0 ~(\bar{ \gamma }_{t}\neq 1), $$

where $\bar{\gamma }_{t}$ is the agent’s coefficient of relative risk aversion at time *t*, $l_{t}$ is the leisure rate at time *t*, and *α* measures the contribution of consumption to the agent’s utility. To accommodate the empirical fact that risk aversion tends to increase significantly at retirement, we assume a constant relative risk aversion with a one-off jump at retirement: $\bar{\gamma }_{t}=\bar{ \gamma }_{1}$ for $t< \tau $ and $\bar{\gamma }_{t}=\bar{\gamma }_{2}$ for $t\geq \tau $.

As in Farhi and Panageas [6], we assume that $l_{t}=1$ for$0\leq t< \tau $ and $l_{t}=L>1$ for $t\geq \tau $. Therefore, the post-retirement leisure, which is larger than the pre-retirement leisure, creates an incentive for retirement. If we define $\gamma _{1}\triangleq 1-\alpha (1-\bar{\gamma }_{1})$ and $\gamma_{2}\triangleq 1-\alpha (1-\bar{\gamma }_{2})$ then (2.2) can be rewritten as

$$ u(c_{t},l_{t})=\frac{c_{t}^{1-\gamma_{1}}}{1-\gamma_{1}}, \quad \mbox{for }0\leq t< \tau\quad \mbox{and}\quad u(c_{t},l_{t})=L^{\gamma_{2}-\bar{\gamma }_{2}} \frac{c_{t}^{1-\gamma_{2}}}{1-\gamma_{2}}, \quad \mbox{for } t\geq \tau . $$

Suppose that the economic agent is allowed to invest in the financial market that consists of two investment opportunities: a riskless asset $M_{t}$ with price process $dM_{t}/M_{t}=r\,dt$, where *r* is the constant rate of return of the riskless asset; and a risky asset $S_{t}$ with price process $dS_{t}/S_{t}=\mu \,dt+\sigma dB_{t}$, where *μ* is the constant rate of return of the risky asset, *σ* is the constant volatility of the risky asset, and $B_{t}$ is the standard Brownian motion on a probability space $(\Omega ,\mathcal{F}, \mathbb{P})$. Denote by $\{ \mathcal{F}_{t} \} _{t \geq 0}$ the $\mathbb{P}$-augmentation of the filtration generated by the standard Brownian motion $\{ B_{t} \} _{t \geq 0}$. Then we can define a portfolio process $\boldsymbol{\pi }\triangleq \{ \pi_{t} \} _{t\geq 0}$, i.e., the amount of money invested in the risky asset, which is a measurable process that is adapted to $\{ \mathcal{F}_{t} \}_{t \geq 0}$ and that satisfies $\int_{0}^{t} \pi^{2}_{s}\,ds<\infty$, for all $t\geq 0$ a.s. A consumption rate process $\mathbf{c}\triangleq \{ c_{t} \} _{t \geq 0}$ in (2.1) is a measurable nonnegative process that is adapted to $\{ \mathcal{F}_{t} \} _{t \geq 0}$ and satisfies $\int_{0}^{t} c_{s}\,ds<\infty$, for all $t\geq 0$ a.s. The retirement time *τ* is an $\mathcal{F}_{t}$-stopping time.

Denote by $I_{1}$ and $I_{2}$ the labor income while working andretirement income (e.g., pension benefits), respectively. Then the agent’s wealth level process $X_{t}$ at time *t* is given by

$$ dX_{t}= \bigl[ rX_{t}+\pi_{t}(\mu -r)-c_{t}+I_{1} \mathbf{1}_{\{0\leq t < \tau \}}+I_{2} \mathbf{1}_{\{t\geq \tau \}} \bigr]\,dt+\pi_{t} \sigma dB _{t}. $$

## The optimization problems and the solutions

The purpose of our study is to examine combined effects of the subsistence consumption and risk aversion change at retirement on the optimal policies of an economic agent. We first obtain the value function and the optimal policies when the economic agent is retired and then obtain those when she is working, by utilizing the smooth-pasting condition of post-retirement value function and pre-retirement value function at the *retirement wealth level*.

### Assumption 3.1

Throughout this paper, we assume that

$$ K_{1}\triangleq r+\frac{\rho -r}{\gamma_{1}}+\frac{\gamma_{1}-1}{2 \gamma_{1}^{2}} \theta^{2}>0,\qquad K_{2}\triangleq r+\frac{\rho -r}{\gamma _{2}}+ \frac{\gamma_{2}-1}{2\gamma_{2}^{2}}\theta^{2}>0, $$

where $\theta \triangleq {(\mu -r)}/{\sigma }$ is the market price of risk. This assumption guarantees that the economic agent’s optimization problems are well defined.

### Post-retirement optimization problem

Firstly, we investigate the value function and optimal consumption and portfolio strategies after retirement. Let us call a pair of control $(\mathbf{c},\boldsymbol{\pi } )$ *admissible* at initial capital $x> (R_{2}-I _{2})/r$, if $X_{t}> (R_{2}-I_{2})/r$ and $c_{t}\geq R_{2}$ for $\tau < t<\infty $. To guarantee $c_{t}\geq R_{2}$ for $\tau < t<\infty $, wealth of the retiree needs to be larger than the discounted value of the minimum consumption stream net of the discounted value of the income stream on an infinite horizon. Therefore, we require $X_{t}> (R_{2}-I_{2})/r$ at any $t\geq \tau $ for the retiree. Let $\widetilde{\mathcal{A}}(x)$ denote the set of all admissible pairs at *x*. The retiree‘s optimization problem is to find the value function

3.1 $$ V_{p}(x)\triangleq \max_{(\mathbf{c},\boldsymbol{\pi } )\in \widetilde{\mathcal{A}}(x)} \mathbb{E} \biggl[ L^{\gamma_{2}-\bar{\gamma }_{2}} \int_{0}^{\infty }e^{-\rho t}\frac{c _{t}^{1-\gamma_{2}}}{1-\gamma_{2}}\,dt \biggr] , $$

subject to

$$\begin{aligned} dX_{t}= \bigl[ rX_{t}+\pi_{t}(\mu -r)-c_{t}+I_{2} \bigr]\,dt+\pi_{t} \sigma dB_{t},\quad X_{t}> (R_{2}-I_{2})/r, \end{aligned}$$

for $t>0$. $\rho >0$ is the agent’s subjective discount rate.

#### Definition 3.1

Let $n_{1}$ and $n_{2}$ be the two roots to the following equation:

$$ \frac{1}{2}\theta^{2} n^{2}+ \biggl( \rho -r+ \frac{1}{2}\theta^{2} \biggr) n-r=0, $$

such that $n_{1}>0$ and $n_{2}<-1$. Similarly, let $m_{1}$ and $m_{2}$ be the roots to the following equation:

$$ r m^{2}-\biggl(\rho +r+\frac{1}{2}\theta^{2}\biggr)m + \rho =0, $$

such that $m_{1}>1$, $0< m_{2}<1$.

#### Proposition 3.1

*The value function* $V_{p}(x)$ *defined* (3.1) *is strictly concave and strictly increasing on* $x\in ((R_{2}-I_{2})/r, \infty )$.

#### Proof

If we follow similar lines to the proof of Proposition 2.1 in Zariphopoulou [24], we arrive at the results. □

Using the dynamic programming method, we obtain the value function $V_{p}(\cdot )\in C^{2}((R_{2}-I_{2})/r, \infty )$ and the related optimal consumption and portfolio.

#### Proposition 3.2

$$ V_{p}(x)=L^{\gamma_{2}-\bar{\gamma }_{2}} \textstyle\begin{cases} {A_{p} ( x-\frac{R_{2}-I_{2}}{r} ) ^{m_{2}}+\frac{R_{2}^{1- \gamma_{2}}}{\rho (1-\gamma_{2})}},&\textit{for } (R_{2}-I_{2})/r< x< \tilde{x}_{2} , \\ {B_{p}\frac{r-\frac{1}{2}\theta^{2} n_{1}}{\rho }\zeta^{-\gamma_{2}(n_{1}+1)}+\frac{\zeta^{1-\gamma_{2}}}{K_{2}(1- \gamma_{2})}},&\textit{for } x\geq \tilde{x}_{2}, \end{cases} $$

*where*

$$ B_{p}=\frac{ ( \frac{m_{2}-1}{\gamma_{2}}+1 ) \frac{1}{K_{2}}- \frac{1}{r}}{(m_{2}-1)n_{1}-1}R_{2}^{\gamma_{2} n_{1} +1}, \qquad \tilde{x} _{2}=B_{p} R_{2}^{-\gamma_{2} n_{1}}+ \frac{R_{2}}{K_{2}}- \frac{I_{2}}{r}, $$

*and*

$$ A_{p}=\frac{1}{m_{2}} \biggl( \tilde{x}_{2}- \frac{R_{2}-I_{2}}{r} \biggr) ^{1-m_{2}} R_{2}^{-\gamma_{2}}. $$

*For* $x\geq \tilde{x}_{2}$, *ζ* *is determined by the following algebraic equation*:

$$ x=B_{p} \zeta^{-\gamma_{2} n_{1}}+\frac{\zeta }{K_{2}}- \frac{I_{2}}{r}, $$

*which is the relationship between the optimal consumption rate* *ζ* *and the wealth level x*. *The optimal consumption and portfolio pair* $(\mathbf{c}^{p,*},\boldsymbol{\pi }^{p,*})$ *for the retiree is given by*

$$ c_{t}^{p,*}= \textstyle\begin{cases} R_{2},&\textit{for }(R_{2}-I_{2})/r< X_{t}< \tilde{x}_{2}, \\ \zeta_{t},&\textit{for }X_{t}\geq \tilde{x}_{2}, \end{cases} $$

*and*

$$ \pi_{t}^{p,*}= \textstyle\begin{cases} {\frac{\theta }{\sigma }\frac{1}{1-m_{2}} ( X_{t}-\frac{R_{2}-I _{2}}{r} ) },&\textit{for }(R_{2}-I_{2})/r< X_{t}< \tilde{x}_{2}, \\ {\frac{\theta }{\sigma \gamma_{2}} ( -\gamma_{2} n_{1} B_{p} \zeta _{t}^{-\gamma_{2} n_{1}}+\frac{\zeta_{t}}{K_{2}} ) },&\textit{for }X_{t}\geq \tilde{x}_{2}, \end{cases} $$

*where *$\zeta_{t}$ *is determined by the following algebraic equation*:

$$ X_{t}=B_{p} \zeta_{t}^{-\gamma_{2} n_{1}}+ \frac{\zeta_{t}}{K_{2}}-\frac{I _{2}}{r}. $$

#### Proof

The relevant Bellman equation for the value function $V_{p}(x)$ is given by

3.2 $$ \rho V_{p}(x)=\max_{c\geq R_{2}, \pi } \biggl[ \bigl\{ rx+\pi (\mu -r)-c+I _{2} \bigr\} V_{p}'(x)+ \frac{1}{2}\sigma^{2}\pi^{2} V_{p}''(x)+L^{ \gamma_{2}-\bar{\gamma }_{2}} \frac{c^{1-\gamma_{2}}}{1-\gamma_{2}} \biggr] . $$

If the subsistence consumption constraint $c_{t}\geq R_{2}$ does not bind, the first order conditions (FOCs) yield the optimal consumption $c^{*}=c^{p,*}$ and portfolio $\pi^{*}=\pi^{p,*}$ (to simplify the notation we drop the superscript *p*) as follows:

$$ c^{*}=L^{\frac{\gamma_{2}-\bar{\gamma }_{2}}{\gamma_{2}}}\bigl(V'_{p}(x) \bigr)^{-\frac{1}{ \gamma_{2}}},\qquad \pi^{*}=-\frac{\theta }{\sigma } \frac{V'_{p}(x)}{V''_{p}(x)}. $$

But due to the subsistence consumption constraint $c_{t}\geq R_{2}$, there exists $\tilde{x}_{2}$ such that

3.3 $$ c^{*}=R_{2},\qquad \pi^{*}=- \frac{\theta }{\sigma }\frac{V'_{p}(x)}{V''_{p}(x)},\quad \mbox{for }(R_{2}-I_{2})/r< x< \tilde{x}_{2}, $$

and

3.4 $$ c^{*}=L^{\frac{\gamma_{2}-\bar{\gamma }_{2}}{\gamma_{2}}}\bigl(V'_{p}(x) \bigr)^{-\frac{1}{ \gamma_{2}}},\qquad \pi^{*}=-\frac{\theta }{\sigma } \frac{V'_{p}(x)}{V''_{p}(x)},\quad \mbox{for } x\geq \tilde{x}_{2}. $$

For $(R_{2}-I_{2})/r< x<\tilde{x}_{2}$, plugging (3.3) into (3.2) yields

3.5 $$ \rho V_{p}(x)=(rx-R_{2}+I_{2})V'_{p}(x)- \frac{1}{2}\theta^{2} \frac{(V'_{p}(x))^{2}}{V''_{p}(x)}+L^{\gamma_{2}-\bar{\gamma }_{2}} \frac{R _{2}^{1-\gamma_{2}}}{1-\gamma_{2}}. $$

We can obtain the general solution to the equation (3.5) as follows:

3.6 $$ V_{p}(x)=c_{1} \biggl( x-\frac{R_{2}-I_{2}}{r} \biggr) ^{m_{1}}+c_{2} \biggl( x-\frac{R _{2}-I_{2}}{r} \biggr) ^{m_{2}}+L^{\gamma_{1}-\gamma }\frac{R_{2}^{1- \gamma_{2}}}{\rho (1-\gamma_{2})}, $$

for some constants $c_{1}$ and $c_{2}$. If $c_{1}=0$ and $c_{2}>0$, $V_{p}(x)$ is a concave function. Therefore, we proceed with $c_{1}=0$ and will see that $c_{2}>0$. The optimal portfolio is given by

$$ \pi_{t}^{*}=\frac{\theta }{\sigma }\frac{1}{1-m_{2}} \biggl( X_{t}-\frac{R _{2}-I_{2}}{r} \biggr) . $$

For $x\geq \tilde{x}_{2}$, by the first order conditions (FOCs) (3.4), the Bellman equation (3.2) for $V_{p}(x)$ can be rewritten as

3.7 $$ \rho V_{p}(x)=(rx+I_{2})V'_{p}(x)- \frac{1}{2}\theta^{2} \frac{(V'_{p}(x))^{2}}{V''_{p}(x)}+\frac{\gamma_{2}}{1-\gamma_{2}}L ^{\frac{\gamma_{2}-\bar{\gamma }_{2}}{\gamma_{2}}}\bigl(V'_{p}(x)\bigr)^{1-\frac{1}{ \gamma_{2}}}. $$

By the strict concavity of $V_{p}(x)$, if we write $w(x)\triangleq V'_{p}(x)$, we can define a function *F* such that $F(w(x))=x+I_{2}/r $ with the identities

3.8 $$ F'(w)V''_{p}(x)=1, \qquad F''(w) \bigl(V''_{p}(x) \bigr)^{2}+F'(w)V'''_{p}(x)=0. $$

By differentiating (3.7) with respect to *x* and using (3.8), we arrive at the following linear ordinary differential equation:

3.9 $$ \frac{1}{2}\theta^{2} w^{2} F''(w)+\bigl(\theta^{2}+\rho -r \bigr)wF'(w)-rF(w)=-L ^{\frac{\gamma_{2}-\bar{\gamma }_{2}}{\gamma_{2}}}w^{-\frac{1}{\gamma _{2}}}. $$

The general solution to the equation (3.9) is given by

$$ F(w)=d_{1} w^{n_{1}}+d_{2} w^{n_{2}}+ \frac{L^{\frac{\gamma_{2}-\bar{ \gamma }_{2}}{\gamma_{2}}}}{K_{2}}w^{-\frac{1}{\gamma_{2}}}, $$

for some constants $d_{1}$ and $d_{2}$. If we write $\zeta \triangleq w^{-\frac{1}{\gamma_{2}}}L^{\frac{\gamma_{2}-\bar{\gamma }_{2}}{\gamma _{2}}}$, which is the optimal consumption from (3.4), we obtain

3.10 $$ x=F\bigl(w(x)\bigr)-\frac{I_{2}}{r}=\tilde{d}_{1} \zeta^{-\gamma_{2} n_{1}} + \tilde{d}_{2}\zeta^{-\gamma_{2} n_{2}}+ \frac{\zeta }{K_{2}}-\frac{I _{2}}{r}, $$

where $\tilde{d}_{1}=d_{1}L^{(\gamma_{2}-\bar{\gamma }_{2})n_{1}}$ and $\tilde{d}_{2}=d_{2}L^{(\gamma_{2}-\bar{\gamma }_{2})n_{2}}$. From (3.7), the value function $V_{p}(x)$ is given by

$$\begin{aligned} V_{p}(x)=\frac{r-\frac{1}{2}\theta^{2} n_{1}}{\rho }\tilde{\tilde{d}} _{1} \zeta^{-\gamma_{2}(n_{1}+1)}+\frac{r-\frac{1}{2}\theta^{2} n_{2}}{ \rho }\tilde{\tilde{d}}_{2} \zeta^{-\gamma_{2}(n_{2}+1)}+\frac{L^{ \gamma_{2}-\bar{\gamma }_{2}}}{K_{2}(1-\gamma_{2})}\zeta^{1-\gamma _{2}}, \end{aligned}$$

where $\tilde{\tilde{d}}_{1}=d_{1}L^{(\gamma_{2}-\bar{\gamma }_{2})(n _{1}+1)}$ and $\tilde{\tilde{d}}_{2}=d_{2}L^{(\gamma_{2}-\bar{\gamma }_{2})(n_{2}+1)}$. We impose the no blow-up condition of the value function to obtain $d_{2}=0$. From (3.10), the wealth level $x^{*}$ corresponding to the optimal consumption $c^{*}$ and portfolio $\pi^{*}$ is a function $X_{p}(\cdot )$ of the optimal consumption $c^{*}$ such that

3.11 $$ x^{*}=X_{p}\bigl(c^{*}\bigr)= \tilde{d}_{1} c^{*-\gamma_{2} n_{1}}+\frac{c^{*}}{K _{2}}-\frac{I_{2}}{r}, $$

for $x\geq \tilde{x}_{2}$. Then

3.12 $$\begin{aligned}& \tilde{x}_{2}=X_{p}(R_{2})= \tilde{d}_{1} R_{2}^{-\gamma_{2} n_{1}}+\frac{R _{2}}{K_{2}}- \frac{I_{2}}{r}, \end{aligned}$$

3.13 $$\begin{aligned}& V'_{p}(\tilde{x}_{2})= m_{2} c_{2} \biggl( \tilde{x}_{2}- \frac{R_{2}-I _{2}}{r} \biggr) ^{m_{2}-1}=L^{\gamma_{2}-\bar{\gamma }_{2}}R_{2}^{- \gamma_{2}}, \end{aligned}$$

and

3.14 $$ V''_{p}( \tilde{x}_{2})=m_{2}(m_{2}-1)c_{2} \biggl( \tilde{x}_{2}-\frac{R _{2}-I_{2}}{r} \biggr) ^{m_{2}-2}= \frac{1}{F'(L^{\gamma_{2}-\bar{\gamma }_{2}}R_{2}^{-\gamma_{2}})}. $$

From (3.13) and (3.14), we have

3.15 $$ \tilde{x}_{2}=(m_{2}-1)n_{1} \tilde{d}_{1}R_{2}^{-\gamma_{2} n_{1}}-(m _{2}-1) \frac{R_{2}}{\gamma_{2} K_{2}}+\frac{R_{2}}{r}-\frac{I_{2}}{r} . $$

Combining (3.12) and (3.15) yields

$$ \tilde{d}_{1}=\frac{ ( \frac{m_{2}-1}{\gamma _{2}}+1 ) \frac{1}{K_{2}}-\frac{1}{r}}{(m_{2}-1)n_{1}-1}R_{2}^{\gamma _{2} n_{1} +1}=B_{p} $$

and

$$ c_{2}=\frac{1}{m_{2}}L^{\gamma_{2}-\bar{\gamma }_{2}} \biggl( \tilde{x} _{2}-\frac{R_{2}-I_{2}}{r} \biggr) ^{1-m_{2}} R_{2}^{-\gamma_{2}}=L^{ \gamma_{2}-\bar{\gamma }_{2}}A_{p}>0. $$

The optimal portfolio

$$ \pi^{*}_{t}=\frac{\theta }{\sigma \gamma_{2}} \biggl( - \gamma_{2} n_{1} B _{p} \zeta_{t}^{-\gamma_{2} n_{1}}+ \frac{\zeta_{t}}{K_{2}} \biggr) $$

is an immediate consequence of the first order condition (3.4). □

### Pre-retirement optimization problem

With the post-retirement value function $V_{p}(x)$ in hand, we seek to investigate the pre-retirement value function, optimal consumption, portfolio, and retirement policies. A triple $(\mathbf{c},\boldsymbol{\pi } , \boldsymbol{\tau})$ of control is called *admissible* at initial capital $x> (R_{1}-I_{1})/r$, if $X_{t}>(R_{1}-I_{1})/r$ and $c_{t}\geq R_{1}$ for $0\leq t<\tau $. The retirement decision is discretionary, and if the economic agent decides not to retire, then $\tau =\infty $. Therefore, we impose the condition $X_{t}>(R_{1}-I_{1})/r$ for $0\leq t<\tau $, similarly to the case of the retiree. Let $\mathcal{A}(x)$ denote the set of all admissible triples at *x*. Before retirement, an economic agent’s optimization problem is to find the value function

$$ V(x)\triangleq \max_{(\mathbf{c},\boldsymbol{\pi } , \boldsymbol{\tau})\in \mathcal{A}(x)} \mathbb{E} \biggl[ \int_{0}^{\tau }e^{-\rho t}\frac{c_{t}^{1-\gamma _{1}}}{1-\gamma_{1}} \,dt+e^{-\rho \tau }V_{p}(X_{\tau }) \biggr] , $$

with the dynamic budget constraint

$$ dX_{t}= \bigl[ rX_{t}+\pi_{t}(\mu -r)-c_{t}+I_{1} \bigr]\,dt+\pi_{t} \sigma dB_{t},\quad X_{t}> (R_{1}-I_{1})/r. $$

The relevant Bellman equation for the value function $V(x)$ is given by

3.16 $$ \rho V(x)=\max_{c\geq R_{1},\pi } \biggl[ \bigl\{ rx+\pi ( \mu -r)-c+I _{1} \bigr\} V'(x)+\frac{1}{2} \sigma^{2}\pi^{2} V''(x)+ \frac{c^{1- \gamma_{1}}}{1-\gamma_{1}} \biggr] . $$

Following Choi and Shim [4] and Dybvig and Liu [5], we conjecture the existence of the wealth accumulation for retirement, say *retirement wealth level*, *x̄*, which corresponds to the optimal retirement time $\tau^{*}$ such that $\tau^{*} = \inf \{t \geq 0: X_{t}\geq \bar{x}\}$. The following verification theorem enables us to find the pre-retirement value function $V(x)$ and the optimal consumption, portfolio, and retirement time.

#### Theorem 3.1

*Suppose that a strictly concave and strictly increasing function* $v(\cdot )\in C^{2}((R_{1}-I_{1})/r, \bar{x})$ *solves the Bellman equation* (3.16) *and satisfies the smooth*-*pasting* (*continuous differentiability*) *condition with* $V_{p}(x)$ *at* $x=\bar{x}$. *Then* $V(x)=v(x)$ *and the optimal consumption* $c^{*}$ *and portfolio* $\pi^{*}$ *are the maximizer of the Bellman equation* (3.16) *and the optimal retirement time* $\tau^{*}$ *is given by* $\tau^{*} = \inf \{t \geq 0: X_{t}\geq \bar{x}\}$.

#### Proof

The proof follows similar lines to those of the proof of Theorem 4.1 in Lee and Shin [15]. □

#### Theorem 3.2

3.17 $$ V(x)= \textstyle\begin{cases} {A ( x-\frac{R_{1}-I_{1}}{r} ) ^{m_{2}}+\frac{R_{1}^{1-\gamma _{1}}}{\rho (1-\gamma_{1})}},&\textit{for } (R_{1}-I_{1})/r< x< \tilde{x}_{1} , \\ {\frac{r-\frac{1}{2}\theta^{2} n_{1}}{\rho } B_{1} \eta^{-\gamma_{1}(n_{1}+1)}+\frac{r-\frac{1}{2}\theta^{2} n_{2}}{ \rho } B_{2} \eta^{-\gamma_{1}(n_{2}+1)}} \\ \quad {}+ {\frac{\eta^{1-\gamma_{1}}}{K_{1}(1-\gamma_{1})}},&\textit{for }\tilde{x}_{1}\leq x< \bar{x}, \end{cases} $$

*where*

3.18 $$\begin{aligned}& x=B_{1}\eta^{-\gamma_{1}n_{1}}+B_{2} \eta^{-\gamma_{1}n_{2}}+\frac{ \eta }{K_{1}}-\frac{I_{1}}{r},\quad \textit{for } \tilde{x}_{1}\leq x< \bar{x}, \\& B_{1}=\frac{ ( \frac{m_{2}-1}{\gamma_{1}}+1 ) \frac{1}{K_{1}}- \frac{1}{r}}{(m_{2}-1)n_{1}-1}R_{1}^{\gamma_{1} n_{1} +1}. \end{aligned}$$

*Let us define*

$$\begin{aligned} G(x)&\triangleq\frac{1}{2}\theta^{2}(n_{2}-n_{1}) \bigl(B_{1}-L^{-n_{1}(\gamma _{2}-\bar{\gamma }_{2})}B_{p}\bigr)x^{-\gamma_{1} n_{1}}+ \biggl( \frac{\rho }{1- \gamma_{1}}-r+\frac{1}{2}\theta^{2}n_{2} \biggr) \frac{1}{K_{1}}x \\ &\quad {}- \biggl( \frac{\rho }{1-\gamma_{2}}-r+\frac{1}{2}\theta^{2}n_{2} \biggr) \frac{L ^{\frac{\gamma _{2}-\bar{\gamma }_{2}}{\gamma_{2}}}}{K_{2}}x^{\frac{\gamma _{1}}{\gamma _{2}}} + \biggl( r-\frac{1}{2} \theta^{2}n_{2} \biggr) \frac{I_{1}-I_{2}}{r},\quad \textit{for } x>0, \end{aligned}$$

*and* *λ* *be the solution to the equation* $G(x)=0$, *then we have*

$$ B_{2}=2\frac{H(\lambda )}{\theta^{2}(n_{1}-n_{2})}, $$

*where*

$$\begin{aligned}& H(x) \triangleq - \biggl( \frac{\rho }{1-\gamma_{1}}-r+\frac{1}{2}\theta ^{2}n_{1} \biggr) \frac{x^{\gamma_{1} n_{2}+1}}{K_{1}} \\& \hphantom{H(x) \triangleq}{}+ \biggl( \frac{\rho }{1-\gamma_{2}}-r+\frac{1}{2}\theta^{2}n_{1} \biggr) \frac{L ^{\frac{\gamma_{2}-\bar{\gamma }_{2}}{\gamma_{2}}}}{K_{2}}x^{\gamma _{1} n_{2}+\frac{\gamma_{1}}{\gamma_{2}}} + \biggl( r-\frac{1}{2}\theta ^{2}n_{1} \biggr) \frac{I_{2}-I_{1}}{r}x^{\gamma_{1} n_{2}}, \\& \tilde{x}_{1}=B_{1}R_{1}^{-\gamma_{1} n_{1}}+B_{2}R_{1}^{-\gamma_{1} n _{2}}+ \frac{R_{1}}{K_{1}}-\frac{I_{1}}{r}, \\& \bar{x}=B_{1}\lambda ^{-\gamma_{1} n_{1}}+B_{2}\lambda ^{-\gamma_{1} n_{2}}+\frac{\lambda }{K _{1}}-\frac{I_{1}}{r}, \end{aligned}$$

*and*

$$ A=\frac{1}{m_{2}} \biggl( \tilde{x}_{1}-\frac{R_{1}-I_{1}}{r} \biggr) ^{1-m _{2}} R_{1}^{-\gamma_{1}}. $$

#### Proof

From the first order conditions (FOCs) and due to the subsistence consumption constraint $c_{t}\geq R_{1}$, there exists $\tilde{x}_{1}$ such that the optimal consumption $c^{*}=R_{1}$ for $(R_{1}-I_{1})/r\leq x<\tilde{x}_{1}$ and $c^{*}= (V'(x)) ^{-\frac{1}{\gamma_{1}}}$ for $\tilde{x}_{1}\leq x<\bar{x}$. Similar arguments to the proof of Proposition 3.2 enable us to reach the expression for the value function $V(x)$ in (3.17) with (3.18) for some constants *A*, $B_{1}$, and $B_{2}$. Similarly to (3.11), we rewrite (3.18) as

$$ x^{*}=X\bigl(c^{*}\bigr)=B_{1}c^{*-\gamma_{1} n_{1}}+B_{2}c^{*-\gamma_{1} n_{2}}+ \frac{c ^{*}}{K_{1}}-\frac{I_{1}}{r}, $$

so we have

3.19 $$\begin{aligned}& \tilde{x}_{1}=X(R_{1})=B_{1}R_{1}^{-\gamma_{1} n_{1}}+B_{2}R_{1}^{- \gamma_{1} n_{2}}+ \frac{R_{1}}{K_{1}}-\frac{I_{1}}{r}, \end{aligned}$$

3.20 $$\begin{aligned}& V'(\tilde{x}_{1})=m_{2}A \biggl( \tilde{x}_{1}-\frac{R_{1}-I_{1}}{r} \biggr) ^{m_{2}-1}=R_{1}^{-\gamma_{1}}, \end{aligned}$$

3.21 $$\begin{aligned}& V''(\tilde{x}_{1})=m_{2}(m_{2}-1)A \biggl( \tilde{x}_{1}-\frac{R_{1}-I _{1}}{r} \biggr) ^{m_{2}-2}=- \gamma_{1}\frac{R_{1}^{-\gamma_{1}-1}}{X'(R _{1})}. \end{aligned}$$

From (3.19)–(3.21), we have

$$ B_{1}=\frac{ ( \frac{m_{2}-1}{\gamma_{1}}+1 ) \frac{1}{K_{1}}- \frac{1}{r}}{(m_{2}-1)n_{1}-1}R_{1}^{\gamma_{1} n_{1} +1},\qquad A= \frac{1}{m _{2}} \biggl( \tilde{x}_{1}-\frac{R_{1}-I_{1}}{r} \biggr) ^{1-m_{2}} R_{1} ^{-\gamma_{1}}. $$

To determine the free boundary *x̄* and the remaining constants, we use the smooth-pasting condition of $V(x)$ with $V_{p}(x)$ at $x=\bar{x}$. If we define

3.22 $$ {\bar{c}_{l}\triangleq \lim_{t\rightarrow \tau^{*}-}c_{t}^{*}} \quad \mbox{and} \quad \bar{c}_{r}\triangleq c^{p,*}_{\tau^{*}}, $$

then we have

3.23 $$ \bar{x}=X(\bar{c}_{l})=X_{p}( \bar{c}_{r}): B_{1}\bar{c}_{l}^{-\gamma _{1} n_{1}}+B_{2} \bar{c}_{l}^{-\gamma_{1} n_{2}}+\frac{1}{K_{1}} \bar{c}_{l}- \frac{I_{1}}{r}=B_{p} \bar{c}_{r}^{-\gamma_{2} n_{1}}+ \frac{ \bar{c}_{r}}{K_{2}}-\frac{I_{2}}{r}, $$

where $B_{p}$ is from Proposition 3.2. We have

3.24 $$\begin{aligned} V(\bar{x}) &=V_{p}(\bar{x}): \frac{r-\frac{1}{2}\theta^{2} n_{1}}{ \rho }B_{1} \bar{c}_{l}^{-\gamma_{1}(n_{1}+1)}+\frac{r-\frac{1}{2} \theta^{2} n_{2}}{\rho }B_{2} \bar{c}_{l}^{-\gamma_{1}(n_{2}+1)}+\frac{ \bar{c}_{l}^{1-\gamma_{1}}}{K_{1}(1-\gamma_{1})} \\ &=L^{\gamma_{2}-\bar{\gamma }_{2}}\frac{r-\frac{1}{2}\theta^{2} n _{1}}{\rho }B_{p} \bar{c}_{r}^{-\gamma_{2}(n_{1}+1)}+L^{\gamma_{2}-\bar{ \gamma }_{2}} \frac{\bar{c}_{r}^{1-\gamma_{2}}}{K_{2}(1-\gamma_{2})} \end{aligned}$$

and

3.25 $$ V'(\bar{x})=V_{p}'(\bar{x}): \bar{c}_{l}^{-\gamma_{1}}=L^{\gamma_{2}-\bar{ \gamma }_{2}}\bar{c}_{r}^{-\gamma_{2}}. $$

From (3.23)–(3.25), we obtain the following algebraic equation for $\bar{c}_{l}$:

$$\begin{aligned} &\frac{1}{2}\theta^{2}(n_{2}-n_{1}) \bigl(B_{1}-L^{-n_{1}(\gamma _{2}-\bar{\gamma } _{2})}B_{p}\bigr)\bar{c}_{l}^{-\gamma_{1} n_{1}}+ \biggl( \frac{\rho }{1- \gamma_{1}}-r+\frac{1}{2}\theta^{2}n_{2} \biggr) \frac{1}{K_{1}}\bar{c} _{l} \\ &\quad {}- \biggl( \frac{\rho }{1-\gamma_{2}}-r+\frac{1}{2}\theta^{2}n_{2} \biggr) \frac{L ^{\frac{\gamma _{2}-\bar{\gamma }_{2}}{\gamma_{2}}}}{K_{2}}\bar{c}_{l}^{\frac{\gamma _{1}}{\gamma _{2}}} + \biggl( r- \frac{1}{2}\theta^{2}n_{2} \biggr) \frac{I_{1}-I _{2}}{r}=0. \end{aligned}$$

Given $\bar{c}_{l}$, we obtain

$$\begin{aligned} &\frac{1}{2}\theta^{2}(n_{1}-n_{2}) B_{2} \\ &\quad = - \biggl( \frac{\rho }{1- \gamma_{1}}-r+\frac{1}{2} \theta^{2}n_{1} \biggr) \frac{\bar{c}_{l}^{ \gamma_{1} n_{2}+1}}{K_{1}} \\ &\quad \quad {}+ \biggl( \frac{\rho }{1-\gamma_{2}}-r+\frac{1}{2}\theta^{2}n_{1} \biggr) \frac{L ^{\frac{\gamma_{2}-\bar{\gamma }_{2}}{\gamma_{2}}}}{K_{2}}\bar{c}_{l} ^{\gamma_{1} n_{2}+\frac{\gamma_{1}}{\gamma_{2}}} + \biggl( r- \frac{1}{2}\theta^{2}n_{1} \biggr) \frac{I_{2}-I_{1}}{r} \bar{c}_{l}^{ \gamma_{1} n_{2}}. \end{aligned}$$

$\tilde{x}_{1}$ and *x̄* are obtained from (3.19) and (3.23), respectively. □

The following pre-retirement optimal strategies are immediate consequences of Theorem 3.1 and Theorem 3.2.

#### Proposition 3.3

*The optimal strategies* $(\mathbf{c}^{*},\boldsymbol{\pi } ^{*}, \boldsymbol{\tau}^{*})$ *for the economic agent while working are given by*

$$\begin{aligned}& c_{t}^{*}= \textstyle\begin{cases} R_{1},&\textit{for }(R_{1}-I_{1})/r< X_{t}< \tilde{x}_{1} , \\ \eta_{t},&\textit{for }\tilde{x}_{1}\leq X_{t}< \bar{x}, \end{cases}\displaystyle \\& \pi_{t}^{*}= \textstyle\begin{cases} {\frac{\theta }{\sigma }\cdot \frac{1}{1-m_{2}} ( X_{t}-\frac{R _{1}-I_{1}}{r} ) },&\textit{for }(R_{1}-I_{1})/r< X_{t}< \tilde{x}_{1} , \\ {\frac{\theta }{\sigma } ( -n_{1}B_{1}\eta_{t}^{-\gamma_{1} n_{1}}-n _{2}B_{2}\eta_{t}^{-\gamma_{1} n_{2}}+\frac{\eta_{t}}{\gamma_{1} K_{1}} ) },&\textit{for }\tilde{x}_{1}\leq X_{t}< \bar{x}, \end{cases}\displaystyle \end{aligned}$$

*and*

$$ \tau^{*}=\inf \{t\geq 0: X_{t}\geq \bar{x}\}, $$

*where* $\eta_{t}$ *solves the following algebraic equation*:

$$ X_{t}=B_{1}\eta_{t}^{-\gamma_{1}n_{1}}+B_{2} \eta_{t}^{-\gamma_{1}n _{2}}+\frac{\eta_{t}}{K_{1}}-\frac{I_{1}}{r}. $$

## Numerical examples

In this section, we use reasonable parameters to explore some numerical results and their implications. To represent empirical observations that at retirement, consumption drops substantially and relative risk aversion increases significantly, we make the following assumption.

### Assumption 4.1

$1<\bar{\gamma }_{1}<\bar{\gamma }_{2}$.

This assumption was also made in Jang and Lee [10] (see detailed justification therein). By the definitions of $\gamma_{1}$ and $\gamma_{2}$, it easily follows that $1<\gamma_{1}<\gamma_{2}$.

### Lemma 4.1

*Suppose that* $B_{1}>L^{-n_{1}(\gamma _{2}-\bar{\gamma }_{2})}B_{p}$. *Then* $G'(x)=0$ *has a unique solution* $x_{e}\in (0,\infty )$ *and* $G''(x)<0$ *for all* $x>0$. *Furthermore*, *if* $G(x_{e})>0$, $G(x)=0$ *has two distinct roots*.

### Proof

$$\begin{aligned} G'(x) &=-\frac{1}{2}\gamma_{1} n_{1} \theta^{2}(n_{2}-n_{1}) \bigl(B_{1}-L ^{-n_{1}(\gamma _{2}-\bar{\gamma }_{2})}B_{p}\bigr)x^{-\gamma_{1} n_{1} -1} \\ &\quad {}+ \biggl\{ \frac{\rho }{1-\gamma_{1}}- \biggl( r-\frac{1}{2} \theta^{2}n _{2} \biggr) \biggr\} \frac{1}{K_{1}}- \biggl\{ \frac{\rho }{1-\gamma_{2}}- \biggl( r-\frac{1}{2}\theta^{2}n_{2} \biggr) \biggr\} \frac{\gamma_{1}L ^{\frac{\gamma_{2}-\bar{\gamma }_{2}}{\gamma_{2}}}}{\gamma_{2}K_{2}}x ^{\frac{\gamma_{1}}{\gamma_{2}}-1}. \end{aligned}$$

By Definition 3.1, $( r-\frac{1}{2}\theta^{2}n_{2} ) =\rho\frac{n_{2}}{1+n_{2}}>0$, and we see that $\lim_{x \downarrow 0}G'(x)=\infty $ and $\lim_{x \uparrow \infty }G'(x)<0$. On the other hand,

$$\begin{aligned} G''(x) &=\frac{1}{2}\gamma_{1} n_{1}(\gamma_{1} n_{1}+1)\theta^{2}(n _{2}-n_{1}) \bigl(B_{1}-L^{-n_{1}(\gamma _{2}-\bar{\gamma }_{2})}B_{p} \bigr)x^{-\gamma _{1} n_{1} -2} \\ &\quad {}- \biggl\{ \frac{\rho }{1-\gamma_{2}}- \biggl( r-\frac{1}{2} \theta^{2}n _{2} \biggr) \biggr\} \frac{\gamma_{1}(\gamma_{1}-\gamma_{2})L^{\frac{ \gamma_{2}-\bar{\gamma }_{2}}{\gamma_{2}}}}{\gamma_{2}^{2}K_{2}}x^{\frac{ \gamma_{1}}{\gamma_{2}}-2}< 0, \end{aligned}$$

so $G'(x)$ is strictly decreasing. Therefore, $G'(x)=0$ has a unique solution $x_{e}\in (0,\infty )$. The existence of two distinct roots to the equation $G(x)=0$, under the condition that $G(x_{e})>0$, is an easy consequence. □

To examine some examples, we use parameters that satisfy the conditions of Lemma 4.1. For such parameters, we are obliged to solve $G(x)=0$ and choose the appropriate one of the two roots, say $x_{m}$ and $x_{M}$ ($x_{m}< x_{e}< x_{M}$). Similar arguments of the proof of the Proposition 4.2 in Lee and Shin [15] lead us to choose $x_{M}$ as the appropriate root to the equation $G(x)=0$.

**Figure 1:**
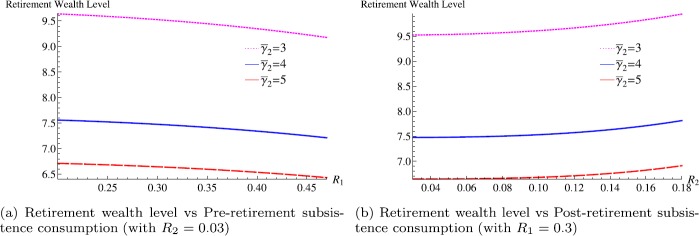
Retirement wealth level and subsistence consumption (parameters: $\rho =0.03$, $r=0.03$, $\mu =0.07$, $\sigma =0.2$, $\alpha =0.5$, $I_{1}=1$, $I_{2}=0.1$, $\bar{\gamma }_{1}=2$, $L=4$)

Firstly, we consider the effects of risk aversion jump and subsistence consumption on the retirement wealth level. Figure 1(a) shows that a strong pre-retirement subsistence consumption may lead to a reduction in the retirement wealth level. With an option to retire, a strong restriction on consumption before retirement would reduce incentive to work, and urge early retirement. In contrast, an economic agent with a strong post-retirement subsistence consumption may be more likely to delay retirement as in Fig. 1(b). Both Fig. 1(a) and 1(b) say that the retirement wealth level decreases as the magnitude of jump in relative risk aversion increases. This finding implies that an individual with a large risk aversion coefficient after retirement (hence invest more in the riskless asset) needs to accumulate less wealth for retirement.

**Figure 2:**
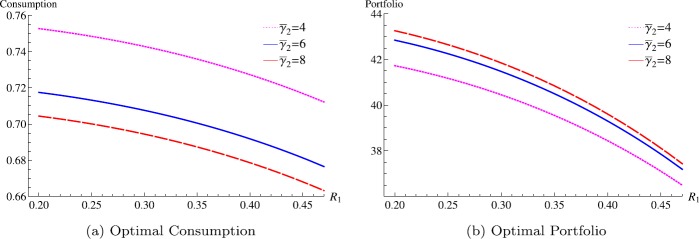
Combined effects of risk aversion change and subsistence consumption on pre-retirement optimal consumption and portfolio at the wealth level 4 (parameters: $\rho =0.03$, $r=0.03$, $\mu =0.07$, $\sigma =0.2$, $\alpha =0.5$, $I_{1}=1, I_{2}=0.1$, $R_{2}=0.03, \bar{\gamma }_{1}=2$, $L=4$)

Figure 2(a) and 2(b) illustrate the optimal consumption and portfolio while working with respect to pre-retirement subsistence consumption and risk aversion change. Because a strong pre-retirement subsistence consumption alone or together with a large magnitude of jump in relative risk aversion tends to urge early retirement (as in Fig. 1(a)), an economic agent is likely to reduce her consumption and increase investment in the risky asset to reach the retirement wealth level earlier. An incentive to increase investment in the risky asset induced by a desire for early retirement competes with the effect of pre-retirement subsistence consumption on investment in the risky asset (a strong pre-retirement subsistence consumption alone seems to reduce investment in the risky asset), and the latter prevails in the case shown in Fig. 2(b).

**Figure 3:**
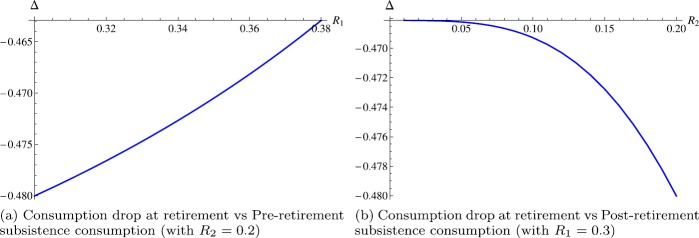
Consumption drop at retirement and subsistence consumption (parameters: $\rho =0.03$, $r=0.03$, $\mu =0.07$, $\sigma =0.2$, $\alpha =0.5$, $I_{1}=1$, $I_{2}=0.5$, $\bar{\gamma }_{1}=2$, $\bar{\gamma }_{2}=5$, $L=4$)

By means of our model, we explore the consumption drop at retirement which has been well reported in the literature. We define the *consumption drop at retirement* Δ by

$$\begin{aligned} {\Delta \triangleq (\bar{c}_{r}-\bar{c}_{l})/ \bar{c}_{l}}=\bar{c}_{l}^{\frac{\gamma _{1}-\gamma _{2}}{\gamma _{2}}} L^{\frac{\gamma_{2}-\bar{\gamma }_{2}}{\gamma _{2}}}-1, \end{aligned}$$

where $\bar{c}_{l}$ and $\bar{c}_{r}$ are from (3.22). It is worthwhile to emphasize that if there is no risk aversion change at retirement, i.e., $\bar{\gamma }_{1}=\bar{\gamma }_{2}$, then consumption drop at retirement Δ is not affected by subsistence consumption $R_{1}$ and $R_{2}$ at all. Figure 3(a) and 3(b) exhibit the effects of subsistence consumption on consumption drop at retirement. Decreasing pre-retirement subsistence consumption deepens consumption drop at retirement, while increasing post-retirement subsistence consumption has an effect in the same direction. Although empirical evidence consistent with such findings is rare, we interpret them as demonstrating that a relatively weak pre-retirement or relatively strong post-retirement restriction on consumption may intensify consumption drop at retirement.

## Conclusion

This paper aims to quantify the effects of subsistence consumption and risk aversion change at retirement on optimal consumption, portfolio, and retirement. Lifetime relative risk aversion is assumed to be constant and to make a one-off jump at retirement. A Cobb–Douglas utility function, which is appropriate for capturing consumption drop at retirement, is employed. The main findings from our analytic solution are as follows. The wealth accumulation for retirement decreases as pre-retirement subsistence consumption increases, and increases as post-retirement subsistence consumption increases. Regardless of the subsistence consumption, a large magnitude of jump in relative risk aversion tends to urge retirement. An interesting finding is that subsistence consumption constraints can affect consumption drop at retirement if the relative risk aversion changes at retirement. Whereas post-retirement subsistence consumption may intensify consumption drop at retirement, pre-retirement subsistence consumption weakens it. We also found that increase in pre-retirement subsistence consumption may lead to decrease in both consumption and investment in the risky asset. In contrast, the magnitude of the jump in relative risk aversion may lead the economic agent to decrease consumption, but to raise investment in the risky asset.

Other than imposing subsistence consumption constraint, considering an incomplete market, for example unhedgeable income risk, is a way to increase realism in investigating lifetime optimal consumption, portfolio, and retirement rules. In this respect, to incorporate income risk in the present paper is a meaningful future research.
